# Linking physical activity to workers’ low back pain, back health, and theory-based psychological variables: study protocol of the *workHealth* intensive longitudinal observational study

**DOI:** 10.1186/s12889-025-21873-7

**Published:** 2025-03-13

**Authors:** Lea O. Wilhelm, Nina Lederle, Lotte-Eleonora Diering, Lara Thiel, Sabine Hahn, Antje Reschke, Greta M. Steckhan, Hendrik Schmidt, Lena Fleig

**Affiliations:** 1 Department of Psychology, Health Psychology and Behavioral Medicine, Medical School Berlin, Berlin, Germany; 2 Department of Education and Psychology, Health Psychology, Freie Universität Berlin, Berlin, Germany; 3 Julius Wolff Institut, Berlin Institute of Health at Charité at Universitätsmedizin Berlin, Berlin, Germany

**Keywords:** Leisure-time physical activity, Sedentary behavior, Workplace, Accelerometer, Back posture, Health action process approach, Fear avoidance model of pain, Physical activity-mediated demand-control model, Back health behavior model, Prevention of low back pain

## Abstract

**Background:**

The high prevalence of low back pain (LBP) in today’s working population and its substantial impact on quality of life call for preventive and sustainable strategies. Physical activity (PA), especially during leisure-time (LTPA), has been proposed as one of the few promising, active preventive measures against the onset of LBP. This is the protocol for the prospective observational study *workHealth* that aims to describe the patterns of PA among workers (including back posture and movement), examine the link between PA, LBP and back health and identify theory-based psychological determinants of LTPA.

**Methods:**

The proposed study is a longitudinal observational study taking place over 2 months with an intensive-longitudinal ecological momentary assessment (EMA) phase. A sample of 252 adults will be recruited from two working populations, sedentary workers, and manually working physiotherapists. At baseline, participants complete a self-report questionnaire and undergo an objective measurement of their back posture and mobility. At 2, 4, and 8 weeks after baseline, participants fill in the follow-up questionnaires. Starting at baseline, participants will also wear an accelerometer and will complete three daily questionnaires for the following 14 days. In addition to the main study, a sub-sample of 8 observational *N*-of-1 studies will have an extended EMA phase of 68 days and a data-driven exit interview. The primary outcome is moderate-to-vigorous LTPA. Data will be analyzed using regression and multi-level models. For the *N*-of-1 studies, a mixed-methods approach will be used including dynamic regressions.

**Discussion:**

Rather than solely examining LBP from a pathological perspective, *workHealth* is one of the first studies to investigate psychological, behavioral and biomechanical risk factors *and* protective resources against LBP. The study will offer insight into theory-based, psychological determinants of LTPA and its relationship to both low back pain and back health. Between-person and within-person level analyses will provide insight on group comparisons of average effects and individual patterns of physical activity in daily life, respectively. Understanding these relationships can inform future behavioral interventions and ultimately contribute to prevention efforts against LBP and the promotion of back health.

**Trial registration:**

German Clinical Trials Register DRKS00025296|| https://drks.de/|| Registration date 28/06/2021

**Supplementary Information:**

The online version contains supplementary material available at 10.1186/s12889-025-21873-7.

## Background

Low back pain (LBP) is a highly prevalent health problem in the adult population and is the leading cause for years lived with disability worldwide [1]. In 2020, LBP affected 619 million people worldwide and is expected to affect 843 million people by 2050 [1]. In Germany, 52.9% of adults experienced LBP over the last year [2]. Overall, 15.5% of German adults experience chronic back pain [2] that lasts three months or longer and occurs almost every day. Despite its high prevalence and cause of work-related disability [3], the aetiology, treatment and prevention of LBP and chronic LBP remain unclear. Recent research suggests that the disease burden of LBP could be reduced by more than one-third if modifiable, behavior-related risk factors, such as elevated body mass index (BMI), and behavioral risk factors, such as smoking, occupational physical activity (e.g., bending, lifting, awkward postures), and sedentary behavior (e.g., prolonged sitting) could be reduced or eliminated [1]. This highlights the importance of targeting specific health behaviors, particularly physical activity (PA) and sedentary behavior, for the prevention of LBP. Therefore, the present study aims to describe the PA patterns and their association with LBP and back health in a sample of healthy office workers and physiotherapists. To develop future behavioral interventions, we also aim to identify modifiable, psychological predictors of PA based on behavior change theory.

### Physical activity and low back pain

PA is a relevant variable for the prevention of LBP and is recommended as one integral prevention strategy by health guidelines in the treatment of non-specific LBP (e.g., National guideline for the treatment of non-specific back pain) [4]. There is, however, inconsistent and insufficient evidence on which type, intensity and dose of PA is most beneficial for preventing LBP across different population groups. For example, the reviews by Oliveira et al. [5] and Sitthipornvorakul et al. [6] did not find support for the link of PA and LBP among adults. These results might be explained by differences in the PA characteristics across included studies (e.g., type, intensity and duration), heterogeneity of PA measurements (objective vs. self-reports), and the inclusion of only a small number of studies of low quality. In terms of domain-specific PA, occupational physical activity has been identified as a risk factor for LBP [1]. However, results from pooled cohort studies have shown that leisure-time physical activity (LTPA) can serve as a protective resource against LBP [7, 8]. Therefore, one goal of this study is to investigate which intensity and type of PA are the most appropriate for the primary prevention of LBP and the promotion of back health in two different groups of workers: sedentary office workers and physiotherapists.

Previous research indicates that inverted pain ratings are not merely comfort ratings suggesting that pain and comfort are two distinct constructs [9–11]. Recognizing that the absence or a low level of low back pain does not necessarily indicate comfort or good health, the workHealth study introduces a measure of subjective health related to the lower back and will investigate its link with PA.

### Understanding physical activity in workers: integrating theory-based concepts from occupational, health, and pain psychology

**Fig. 1:**
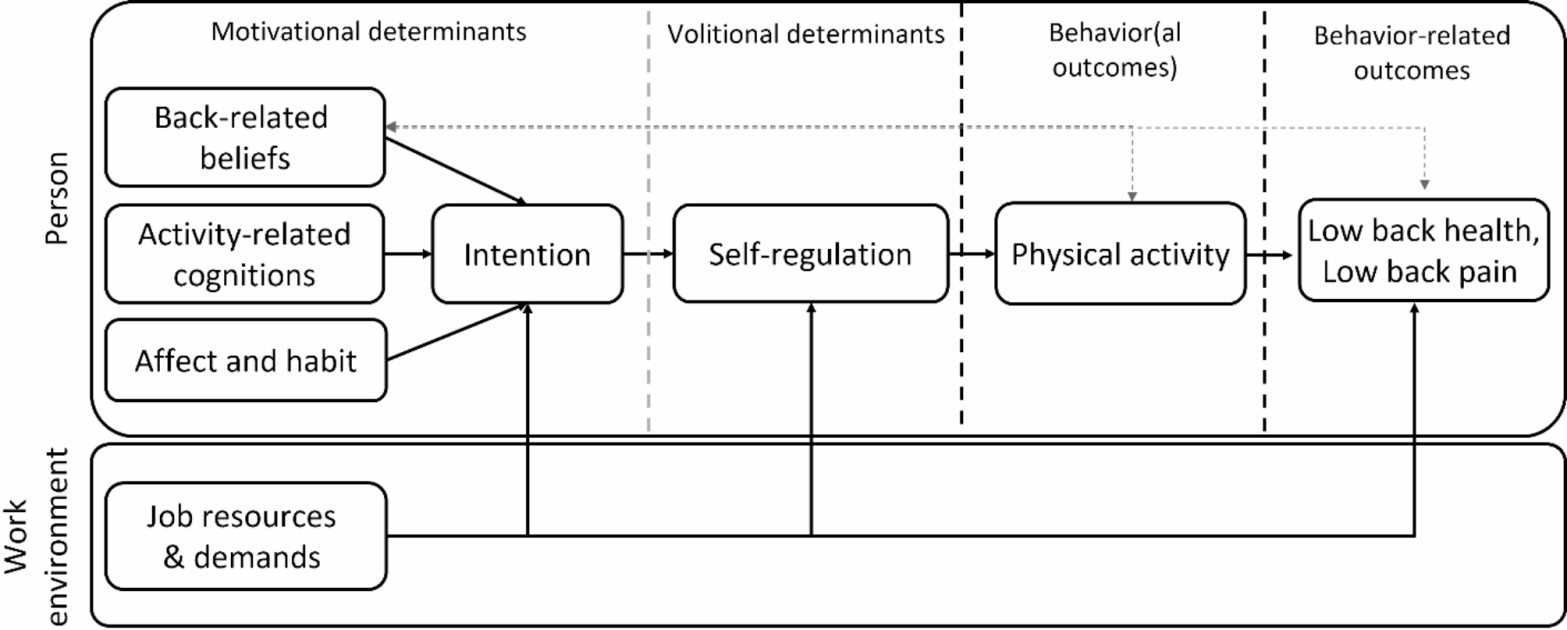
Theoretical framework of the workHealth study: Back Health Behavior Model (BHBM)

Understanding the links between different characteristics of PA, back health and LBP helps to identify target behaviors for future interventions. Theories of behavior change and pain development, on the other hand, can help us to better understand and target the psychological predictors that contribute to the prevention of LBP [12, 13] and the engagement in PA. To do so, the present study draws upon theories from pain (fear avoidance model; avoidance-endurance model of pain) [14–16], occupational (physical activity-mediated Demand-Control model, pamDC) [17] and health psychology (Health Action Process Approach, HAPA; Physical Activity Adoption and Maintenance, PAAM) [18, 19] and integrates these in the Back Health Behavior Model (BHBM; Fig. 1). This model allows for a more nuanced understanding of LTPA adoption in the context of low back pain prevention, acknowledging that LTPA (a) can be understood as a proactive way to cope with LBP (i.e., approach behavior/confrontation) which is partially determined by individuals’ beliefs about their back and pain, that LTPA is (b) dependent on other life domains, such as the workplace, affecting individuals’ general self-determination [20] and capacity to pursue their goals, and that LTPA is (c) not solely the result of conscious intention but also shaped by implicit processes, such as affective states and habits [21, 22].

### Temporal resolution in prevention research

Previous studies assessing psychological predictors of LTPA and LBP have mainly employed prospective designs across several weeks or months, looking at between-group comparisons of average effects, e.g [23]. Psychological factors that frequently correlate with LTPA (e.g., intention, fear of movement) are often unstable and tend to fluctuate within individuals. These factors should be explored, as they change across time in daily life, e.g [24]. Therefore, in the present study, we also adopt a within-person design to examine individual patterns of LTPA in daily life and to identify the strongest predictors of outcomes.

### Aims and research questions

#### Between-person research questions

This observational study aims to (a) describe the health status of the lower back and PA patterns of working people, to (b) examine the relationship between LBP, back health (i.e., behavior-related outcomes) with PA, and to (c) examine theory-based, psychological predictors of workers’ LTPA. At the macro level, we will describe physical activity patterns in workers’ daily lives in terms of intensity, frequency, duration, and type of activity. At the micro level, we will describe posture and movement of the back using self-reported and objective biomechanical measures. We will analyze the back health and physical activity patterns of people in different work contexts. This includes people who work in a sedentary environment (e.g., office work) and people who do manual work as part of their job, such as physiotherapists.

We hypothesize that individuals from different work contexts will differ in their back health and in their levels of LTPA. We also hypothesize that overall higher mean levels of LTPA will be positively associated with workers’ subjective back health, posture and mobility, and negatively associated with pain. Figure 1 shows the set of theory-based, psychological predictors of LTPA intention and LTPA. Based on the Back Health Behavior Model (Fig. 1) we have the following hypotheses. We hypothesize that overall higher mean levels in activity-related cognitions will be positively associated with LTPA intention, with the exception of risk perception and negative outcome expectancies, for which we expect the opposite. We also hypothesize that overall higher mean levels of back-related beliefs, negative affect, and job demands will be negatively associated with LTPA intention. For positive affect, habit, and job resources, we expect positive associations with LTPA intention. We expect a positive association between overall higher mean levels of LTPA motivation and volitional strategies of self-regulation as well as job resources, and the opposite for job demands. Finally, we hypothesize overall higher mean levels of LTPA to be predicted by volitional strategies of self-regulation. We will also explore whether the relationship between back health and job demands and job resources is mediated by LTPA.

#### Within-person research questions

At the within-person level, we hypothesize that higher-than-usual levels of LTPA are positively linked with low back health. We will examine these associations concurrently (same-day, t) and prospectively (from previous-day, t-1 to next day, t). We also expect individuals to engage in more LTPA than usual on days when their positive affect is higher and their negative affect is lower than usual. On days when individuals experience higher levels of job demands and lower levels of job resources than usual, we expect people to engage in lower levels of LTPA than usual. We will examine these associations concurrently (same-day, t) and prospectively (from previous-day, t-1 to next day, t). We will also describe day-to-day fluctuations in activity-related intention, low back pain, social support, planning, self-efficacy, and plan enactment.

In an additional, observational *N*-of-1 time-series approach including data-prompted interviews, we will examine whether these within-person predictors also hold relevance for a single individual. Qualitatively, we aim to understand the related mindsets, and the struggles, and resources involved in carrying out LTPA. This study expands on previous research by examining not only personal predictors but also the impact of social and work environments on low back health. Furthermore, we investigate strategies individuals use to maintain low back health.

## Methods

### Study design and procedures

**Fig. 2:**
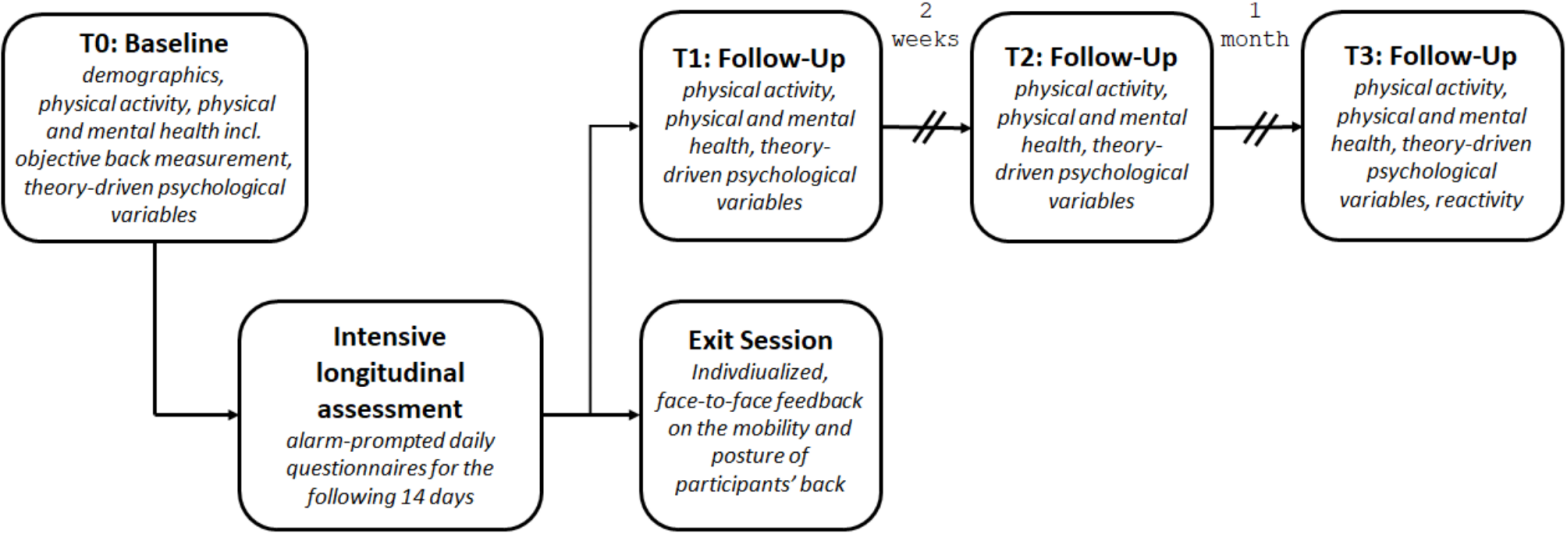
Study design

This study is a prospective longitudinal observational cohort study, taking place over a period of 2 months, with an intensive-longitudinal ecological momentary assessment (EMA) phase (Fig. 2). Participants provide self-report questionnaire data at baseline (Time 0, T0), with subsequent follow-up questionnaires (Time 1–3, long-longitudinal design; LL) collected electronically via an online survey platform (Unipark, Tivian XI GmbH, Cologne, Germany) from a study tablet or computer on-site (T0) or on a personal device at home (e.g., computer, tablet, smartphone; Time 1–3). The study will involve a 1.5 h baseline session at MSB Medical School Berlin or Charité Universitätsmedizin Berlin or participants’ workplace, during which demographics, PA and sedentary behavior, physical and mental health, as well as theory-driven psychological variables will be assessed. During the baseline session, participants will also undergo objective measurements of their back posture and mobility. Participants will then receive training in the time-sampling protocol, the handling of their Android study phone (Nokia 6.3) with the movisensXS app (movisens GmbH) and accelerometer (Move4, movisens GmbH). Starting at T0, participants will wear an accelerometer and will be asked to complete three daily questionnaires on their study smartphone for the following 14 days (intensive-longitudinal design; IL; Fig. 2). Participants will receive three alarm-triggered daily life surveys via the movisensXS app at 8 am (“morning questionnaire”), 3 pm (“after work questionnaire”), and 9 pm (“evening questionnaire”). Participants can delay responding until 3 h after the time prompt, and reminders are sent every 20 min during this window. After this time, the questionnaires will no longer be available to prevent backfilling. Daily life assessments take 2 to 6 min each to complete, resulting in a total of 1.5–4.5 h to complete the diaries over the entire IL phase. Accelerometers will be worn on a belt clip placed at the right hip (i.e., the right anterior iliac crest) and do not require any attention from participants. Participants are instructed to wear the accelerometer during waking hours for 2 weeks and to recharge the battery at least every 5 days. Throughout the study, participants are encouraged to carry out their usual daily activities. The device has no monitor and does not provide activity feedback. Within 2 weeks of the end of the intensive longitudinal phase (1–15 days), participants return their materials (i.e., study phone and accelerometer) and complete a 30-minute exit session to receive feedback on their back posture and mobility. Each participant will be compensated with a minimum of €40 for this study.

A subgroup of interested participants will participate in an observational *N*-of-1 time-series approach [25] over 2.5 months with prospective IL assessments only. Baseline measurements are the same as in the main study including a self-report questionnaire and an objective back posture and mobility measurement, followed by 67 more days of EMA assessments. *N*-of-1 participants will not receive follow-up questionnaires. Within 2 weeks of the end of the IL phase (day 1–68), participants will return their materials (i.e., study phone and accelerometer) and complete a 60-minute exit session to receive feedback on their back and to take part in a data-prompted, qualitative interview [26, 27]. The university’s ethics committee at MSB Medical School Berlin granted approval on 29/03/2021 (approval number MSB-2021/62) for the study, including data management and data protection procedures. Written informed consent will be obtained from each participant prior to enrolment. The pre-registration in the publicly available German Clinical Trials Register (DRKS-ID: DRKS00025296) took place on 28/06/2021. The actual study start date was 22/07/2021 and recruitment continued until the end of the funding period (30/06/2024). All data will be stored on a secure server at the two institutions. Primary data, such as participant-reported outcomes, will be pseudonymized.

### Sample and recruitment

Working adults will be recruited in the greater Berlin area, Germany, through posters and leaflets, email newsletters, intranet postings, research fairs, social media postings and videos, as well as face-to-face presentations to recruit physiotherapists in their workplace. An optional registration website allows participants to leave their contact details. Participants responding to the advertisements will be emailed the participation information and invited for a brief introductory telephone call to explain the procedure, to review inclusion and exclusion criteria, and to schedule a baseline appointment. After receiving verbal and written information, participants provide written informed consent to participate in the study at the start of the baseline session. Participants will have the option to participate in the *N*-of-1 design instead of the main study. Due to the different data protection components (e.g., audio-recording for semi-structured interviews) between the main sample and the *N*-of-1 sub-sample, participants will receive separate information material and written consent forms.

### In- and exclusion criteria

Individuals must be aged between 18 and 64 and working in one of two possible working conditions, either in a predominantly sedentary job (e.g., “office work”) or as physiotherapist (predominantly manual job) for at least 20 h per week, to take part in the study. Individuals should also be able to hear a smartphone-based alarm and be able to read and understand normal German text on a 10-inch tablet and 6-inch smartphone.

Exclusion criteria are: acute and chronic pain [28, 29] (i.e., “pain that persists or recurs for longer than 3 months”), an acute infection, pregnancy, professional, competitive, and top athletes, radicular symptoms, sensory deficits, or neurological diseases, fractures of the spine, diagnosed osteoporosis, strong deformations, or misalignment of the spine (e.g., highly pronounced, structural scoliosis or severe M. Bechterew), recent pelvis operation (3 months post), past spine operations, tumor diseases and bone metastases, inflammation of the spine, spinal stiffening surgery in the past, strong drug therapy (opioids / muscle relaxants / anti-epileptic drugs), an inability to perform the back measurements (max. flexion / extension, and max. lateral bending to the left and right side) as well as current participation in another study. To facilitate the interpretation of back posture and mobility measurements we further exclude persons with a BMI above 29.4 kg/m².

### Benefits and Harms

Due to the non-invasive nature of the back measurements, accelerometer measurements and questionnaires, we did not expect any harm beyond the time commitment of the participants. However, a few of the test positions of the back posture and mobility measurements (particularly leaning backwards and remaining in this position for a few seconds) might cause some sort of fatigue, effort or temporary discomfort for some of the participants. Participants will be compensated (see above), and those who study at the MSB Medical School Berlin in addition to their professional activities will receive additional course credits. Participants will be encouraged to report any adverse effects they may experience during their participation in the intervention over email, text message or by phone call to the study facilitator when they occur (Table 1).

**Table 1:** Variables assessed during the long-longitudinal study period at different time points

Variable	Baseline		2 weeks	4 weeks	8 weeks
**Demographic information**: age, gender, job, children, family status, household members, education, vocational training, mother tongue, citizenship, income, smoking, pregnancy, diseases and disease-related impairment, medication intake, height and weight (to calculate BMI), occupational physical activity, intensity of patient contact (hands-on vs. hands-off; for physiotherapists only), primary place of work/home-office, COVID quarantine	x		-	-	-
**Behavioral outcomes**					
**Physical activity and sedentary behavior**: adapted International Physical Activity Questionnaire (IPAQ) long version	x		x	x	x
**Physical activity and sedentary behavior (objective)**: Move4 accelerometer	Continuous assessment			-	-
**Back posture and mobility**: Back Posture, Movement and Mobility (B-PMM) self-report questionnaire	x			-	-
**Back posture and mobility (objective)**: Medimouse IDAG	x			-	-
**Behavior-related outcomes with respect to the lower back**					
**Low back health**: single item adapted from the Veterans Rand 12-item Health Survey (VR-12)	x		x	x	x
**Back pain**: Chronic Pain Grade Questionnaire, disability due to back pain (Roland-Morris Disability Questionnaire)	x		x	x	x
**General mental and physical health**: Health-related Quality of Life (VR-12), depressive symptoms and anxiety (Patient Health Questionnaire-4, PHQ-4)	x		x	x	x
**Theory-driven psychological variables**					
**Activity-related cognitions**: outcome expectancies, risk perception, self-efficacy, intention, action and coping planning, action control, social support, habit strength (Self-report Behavioral Automaticity Index)	x		x	x	x
**Back-related beliefs**: fear of movement (Tampa Scale for Kinesiophobia), pain catastrophizing (Pain Catastrophizing Scale), fear avoidance beliefs for work-related physical activity (single item from Fear avoidance Beliefs Questionnaire)	x		-	-	-
**Job demands and resources**: job control (Instrument for Stress-Oriented Task Analysis), time pressure (Instrument for Stress-Oriented Task Analysis), recovery experience (Recovery Experience Questionnaire), organizational health climate towards physical activity (Organisational Health Behavior Climate Scale), work environment	x		x	x	x
**Affect**: Positive and Negative Affect Schedule	x		x	x	x
**General self-control**: Brief State Self-Control Capacity Scale	x		x	-	-
**Feedback from participants**					
Reactivity (to sensor, to smartphone-based EMA assessment), interference of study participation with daily life	-		x	-	-
Representativeness of intensive longitudinal phase for daily life	-		x	-	-
Feedback from participants with regard to study participation in general	-		-	-	x
Adverse effects	At any time				

### Measurements

#### Measures for the long-longitudinal phase

The study measures will be taken at four time points, at baseline (T0), and at three follow-up occasions at 2 (Time 1, T1), 4 (Time 2, T2) and 8 (Time 3, T3; Fig. 2; Table 1) weeks post-baseline. At baseline, participants are asked to self-report a number of key demographic variables, including age, gender, marital status, education, and work status. Time-varying demographics will be reassessed at follow-up including medication intake, quarantine, or working from home.

##### Primary outcome

Our primary outcome for the LL phase will be *LTPA*. We operationalize LTPA as minutes spent in moderate-to-vigorous physical activity (MVPA) outside of working hours. An adapted version of the long International Physical Activity Questionnaire (IPAQ) [30] is used to determine LTPA over the past 7 days. The original 27-item instrument assesses different intensities of activities in four domains (job, transport, domestic, and leisure) and time spent sitting. We adapted the instructions for domestic tasks to explicitly include caring for the family (e.g., looking after children, elderly people). For descriptive purposes, we also assess the type of domestic or leisure activity in each case (“What was the activity?“, translated from German). Validation of the German-language instrument by Wanner et al. [30] showed moderate relationships with objectively-assessed PA data, comparable to other self-report PA instruments. The validity of the IPAQ will also be empirically tested by determining the agreement with the accelerometry data from the present study. The IPAQ will be processed in line with the IPAQ protocol [31]. The primary outcome of LTPA for the LL design encompasses items assessed by the IPAQ from the domain of “leisure”. This covers only part of our definition of LTPA, with transport-related and domestic activities excluded. To aggregate activities of different intensities for LTPA, we will determine each activity’s MET-value (Metabolic Equivalent of Task; moderate activity = 4 MET, vigorous activity = 8 MET, walking = 3.3 MET, cycling = 6 MET), multiply it by the time spent in the activity and frequency per week, and sum all values to create MET-minutes per week from LTPA activities (LTPA MET-minutes/week).

##### Secondary outcomes

Secondary outcomes measured at each time point (baseline, 2, 4, and 8 weeks) will include self-reported sedentary behavior, back posture and mobility, and behavior-related variables with respect to the lower back.

We measure *sedentary behavior* through self-report using three items adapted from the International Physical Activity Questionnaire (IPAQ) [30]. To measure leisure-time sedentary behavior, the item stem “During the past 7 days, how much time did you usually spend sitting…” is followed by (a) “…on a typical weekday during leisure-time (e.g., after work, transport time home)?”, and (b) “…on a typical weekend day during leisure-time?”. For work-time sedentary behavior, the item stem is followed by “…on a typical workday?”. All three elements of sedentary time are answered by open-ended responses to hours and minutes spent sitting.

To assess activity at the micro level of the back, we developed a self-report questionnaire to assess *perceived back posture*,* back mobility*,* and back movement* during work and leisure-time: the Back Posture, Movement and Mobility (B-PMM) self-report questionnaire. The B-PMM self-report questionnaire consists of 13 items, 7 items measuring perceived back posture (e.g., “My back posture is very good.”), 5 items measuring perceived back movement (e.g., “I regularly change my back posture at work [e.g., leaning backwards in my office chair, rotating my shoulders, standing up].”) and 1 item assessing back mobility (“I am very flexible in the back.”). The items can be answered on a 6-point Likert scale (*1 = not true at all* to *6 = extremely true*). For a full German and English item list see Additional file 1. For posture and movement, a mean score of the corresponding items will be calculated. The validity of the B-PMM will also be tested by determining the agreement with the objective back measurement data from the present study.

**Fig. 3:**
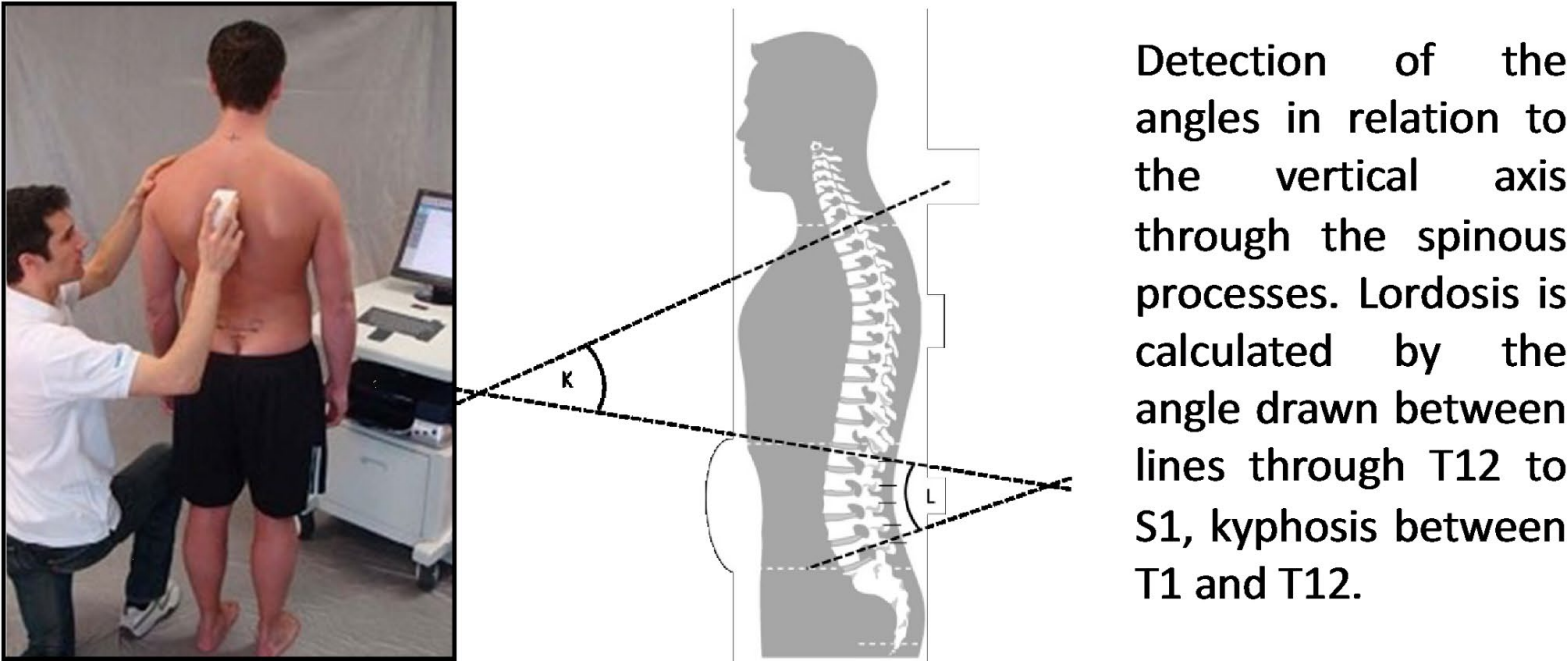
Back posture and mobility measurements using the IDIAG M360^®^ MediMouse

*Back posture and mobility* are measured objectively once at baseline using the IDIAG M360^®^ MediMouse. This device is a hand-held computer-assisted electro-mechanical tool that allows the non-invasive and radiation-free assessment of the spinal shape using two rolling wheels that transfer the spinal contour via Bluetooth to a computer. For this, the tool is guided along the spine on the spinous processes starting at the C7 and ending at the caudal reference point or the top of the anal crease, respectively [32, 33]. The IDIAG M360^®^ MediMouse takes scans in the sagittal and frontal planes of the lumbar and thoracic spine (Fig. 3). After disinfecting the back, the spinous process of the C7 vertebrae is identified by manual palpation. The anatomical landmarks C7 and S3 act as the start and end points of the measurement. In total, participants will be measured in 12 different conditions (six in standing and six in sitting), which will take approximately 20 minutes. The validity and reliability were established in previous studies [33, 34]. The mobee ^®^ spine software (SportMed A.G. SA, Echternach, Luxembourg, version 4.5.0) will be used to process the raw data from the back posture and mobility measurements. For each of the 12 measurement conditions, the lordosis-, kyphosis-, and sacral angles as well as the angles at each spinal segment will be analyzed descriptively for each participant (*M* ± *SD*). In addition, the degree of deviation of the angles from the cohort mean (°), will be analyzed descriptively for each participant. The degree of deviation, which is the difference from the mean, will be defined < 1 *SD*, > 1 *SD* and *> 2 SD* from the mean of the cohort. A ‘back deviation-sum score’ for deviations > 2 SD will be calculated for posture and mobility. The score will range from 0 to 48 for posture (based on 12 test conditions at 4 different angles) and 0–32 for mobility (based on 8 test conditions at 4 different angles). All angles will be normalized to the mean value of the study cohort, which serve as physiological reference value for back posture and mobility. Both absolute scores and difference scores exceeding 2 standard deviations per measurement condition will be utilized as independent variables for multivariate analyses, and sum scores will be examined.

We use multiple indicators to assess behavior-related outcomes with respect to the lower back (Table 1). *Subjective health with regard to the lower back* of participants will be assessed with a single item adapted from the Veterans Rand 12-Item Health Survey (VR-12) [35–37]. We ask participants to rate the question “How would you rate the health of your lower back?” using a Likert scale from 1 (*poor*) to 5 (*excellent*). In line with the time frame of the IPAQ, we instruct participants to think about the past 7 days when answering the item. *General health-related quality of life* (HrQoL) is measured with a modified Veterans Rand 12-Item Health Survey (VR-12) [35–37]. The modifications relate to the time frame and the inverted display of the response options for subjective health. These are displayed from 1 (*poor*) to 5 (*excellent*). In addition, we use the Patient Health Questionnaire (PHQ-4) [38] to screen participants for symptoms of *depression and anxiety*.

We use the Chronic Pain Grade Questionnaire (CPGQ) [39] to assess potential *low back pain intensity and disability*. We specifically instruct participants to think about their lower back when answering the items. Adjustments for pain location have been made before for back pain [40]. For the CPGQ, for exploratory purposes, we added an item specifically tailored to our research question: “In the past 7 days, to what extent has your back pain interfered with your leisure-time physical activity?”. To assess potential *disability due to back pain* we use the Roland-Morris Disability Questionnaire (RMDQ) [41].

##### Determinants of behavior-related and behavioral outcomes

Theory-derived psychological constructs will include self-report measures specific for LTPA, including *risk perception* [42], *outcome expectancies* [42, 43], *intentions*,* self-efficacy*,* action control* [43, 44], *social support* [45–47], *action and coping planning* [48]. *Habit strength* for LTPA will be measured with the Self-Report Behavioral Automaticity Index [49, 50]. Based on the fear avoidance model [15], we will also assess beliefs about the lower back, *fear of movement* using the German version of the Tampa Scale for Kinesiophobia [51, 52] and *pain catastrophizing* using the Pain Catastrophizing Scale [53, 54]. Theory-based job resources will include *job control* (Instrument for Stress-Oriented Task Analysis) [55], *recovery experience* (Recovery Experience Questionnaire) [56], and *organizational health climate* towards physical activity (Organisational Health Behavior Climate Scale) [57]. Time pressure will be assessed with the Instrument for Stress-Oriented Task Analysis [55]. To assess *positive and negative affect*, we use an adapted 10-item short version [58, 59] of the Positive And Negative Affect Schedule (PANAS) [60, 61]. The original item “distressed” was changed to “irritable”, and we added the two negative affect items “sad” and “stressed” (Table 2). *Self-regulatory capacity* is measured with the Brief State Self-Control Capacity Scale [62]. A detailed list of measurements is available on request from the authors.

**Table 2:** Ecological momentary assessment questions asked daily during the intensive-longitudinal study period

Item	Domain	Morning	Afternoon	Evening
**Behavior**				
Have you been physically active during your leisure-time today? *1 (yes) – 2 (no)*; When did you start (time, e.g., 06:30 pm)? *Open answer format (hh: mm)*; What was the activity? *Open answer format*; How long did the activity last? (in min, e.g., 20 min) *Open answer format (min)*	Leisure-time physical activity	-	-	x
Have you carried out your plans from this morning? *1 (not at all true) – 6 (absolutely true)*,* 7 (I did not make any specific plans this morning)*	Plan enactment	-	-	x
Being physically active in my planned situation is something I do automatically. *[Hidden if plan enactment = 7] 1 (not at all true) – 6 (absolutely true)*	Habit strength	-	-	x
**Behavior-related outcomes with respect to the lower back**				
How is your lower back today? *1 (poor) – 5 (excellent)*	Subjective health in relation to lower back	-	-	x
How would you rate your low back pain as it is today? *0 (no pain) – 10 (worst pain imaginable)*; To what extent has your low back pain today interfered with your leisure-time physical activities? *0 (no interference) – 10 (I was unable to carry out any activities)*	Low back pain	-	-	x
**Activity-related variables**				
Today I intend to be physically active in my leisure-time. *1 (not at all true) – 6 (absolutely true)*	Intention	x	-	-
I am confident that today I can manage to be physically active in my leisure-time, even if it is difficult. *1 (not at all true) – 6 (absolutely true)*	Self-efficacy	x	-	-
Today, I received support from my colleagues or supervisor (Item 1)/friends or family (Item 2) with regard to my leisure-time physical activity. *1 (not at all true) – 6 (absolutely true)*	Social support	-	x	-
For today, I have already made a detailed plan on which occasions I can be physically active during my leisure-time. *1 (not at all true) – 6 (absolutely true)*; *[If ≥ 2 (not true)]* What activity(ies) are you planning to do? *Open answer format*; On which occasions (6 pm; in front of the television)? *Open answer format*	Action planning	x	-	-
**Job demands and resources**				
Did you work today? *1 (yes) − 2 (no); [If 1 (yes)]* From when until when did you work today? *From (time*, e.g.,* 9:00 am) ___ Until (time*, e.g.,* 4:00 pm) ___;* Have you taken at least one break of at least 15 min today? *1 (yes) − 2 (no)*; *[If 1 (yes)]* How long was your break? If you took more than one break, think of the longest. *From (time*, e.g.,* 12:00 pm) ___* *Until (time*, e.g.,* 1:00 pm) ___*	Working hours and breaks	-	-	x
Are you still working? *1 (yes) − 2 (no) – 0 (I did not work today)*; *[only for 1 (yes) and 2 (no)]* Are you working from home today? *1 (yes) − 2 (no)*	Work place	-	x	-
How often have you been under time pressure for professional reasons today? *1 (very seldom/never) – 5 (very often/always)*	Time pressure	-	x	-
Were you able to choose the way you did your work today? *1 (very seldom/never) – 5 (very often/always)*	Job control	-	x	-
This evening I didn’t think about my work at all. (Item 1)/ This evening I used the time to relax. (Item 2)/ This evening I did things that challenged me. (Item 3)/ This evening I felt I could decide for myself what to do. (Item 4) *1 (does not apply at all) − 5 (applies completely)*	Recovery experience	-	-	x
**Affect**				
How do you feel at the moment? nervous (Item 1), alert (Item 2), upset (Item 3), scared (Item 4), inspired (Item 5), enthusiastic (Item 6), afraid (Item 7), determined (Item 8), irritable (Item 9), excited (Item 10), stressed (Item 11), sad (Item 12) *1 (very slightly or not at all) – 2 (a little) – 3 (moderately) – 4 (quite a bit) – 5 (extremely)*		x	x	-

#### Measures for the intensive-longitudinal phase: ecological momentary assessment

PA is measured objectively by accelerometer and self-report, sedentary behavior by accelerometer only. Back health and psychological determinants of LTPA will be assessed, including items adapted from previous studies or from longer questionnaires and adapted for EMA purposes.

##### Self-report EMA measures

Participants will be asked to complete self-report EMA measures at a pre-specified time each day. Measures taken at each data collection point in the morning will include *intention*,* self-efficacy* [43, 44], *planning* [48], and *affect* [58, 59]. Measures taken at each afternoon data collection occasion will include *social support* [63, 64], *time pressure*, and *job control*, adapted from [55]. Measures taken at each data collection point in the evening will include LTPA, *plan enactment*, adapted from [65], *habit strength* [50], *low back health*,* LBP intensity* and *disability* [39], *working hours*, *breaks*, and *recovery experience* [66] (Table 2). Each evening, participants are asked how typical their day was and whether anything unusual happened that could have affected their LTPA or accelerometer. In terms of LTPA, participants report whether they did any activity, and if so, what activity they did, when they did it and how long it lasted. This information is used to validate the accelerometer data and to determine the type of LTPA for descriptive purposes (Table 2). At the first follow-up of the LL phase, we also assess reactivity to the IL phase assessment (Table 1).

To obtain an objective measure of LTPA, we use the self-reported work time as a marker for the accelerometry data. By combining these two sources of data, we will be able to distinguish between accelerometer data during work and during non-work time (i.e., leisure-time). The same procedure will be used to obtain a measure for sedentary time during work and leisure-time.

##### Objective EMA measures

For accelerometry we use the Move4 [67] which is a sensor based on triaxial acceleration, gyroscope, barometric air pressure, and temperature. It measures acceleration with a measurement range of ± 16 g and an output rate of 64 Hz, for further specifications, see [68]. Raw data is downloaded from the accelerometers via the SensorManager (movisens GmbH). The accelerometer determines the non-wear time in 30 s intervals [69]. Self-reported data on leisure-/work time information (Table 2, ambulatory assessment, e.g., 9:30 am- 4:30 pm) is checked for plausibility, corrected, and combined with the accelerometer signal by an Octave (GNU Octave, version 7.1.0) script provided by movisens. This enriched raw data is run through the DataAnalyzer software (movisens GmbH, version 1.16.3) to obtain 60 s outputs. Following proprietary algorithms [70], the DataAnalyzer produces physical activity intensity and energy expenditure results based on activity-related regression models, fed by participant characteristics (gender, age, weight, height). Results contain minute-by-minute METs (1 MET equals the energy expenditure at rest), steps, and energy expenditure in calories. MVPA outside of working hours is based on MET-values > 3 MET. Light physical activity is categorized for > 1.5–3 MET. By combination with self-reported work time, LTPA is available on a minute-by-minute basis and will be the main outcome measure of the EMA study. Daily wear time is calculated by subtracting non-wear time from the daily recording time (e.g., 24 h). Following Troiano, Berrigan [71], days with ≥ 10 h of wear time will be considered valid. Participants with ≥ 3 valid days will be included in the data analysis [72]. We will check and potentially control for the initial elevation bias [73].

Sedentary behavior is operationally defined as any behavior in a sitting, reclining, or lying position with ≤ 1.5 MET, excluding sleep [74, 75]. Minute-by-minute sedentary behavior during leisure-time and at work are used as a secondary outcome. To maximise the amount of data available for PA, participants will be reminded to charge their Move4 device every 5 days. If participants agree, we will also contact them via their study smartphone 1 week into the study to see if there are any technical problems with any of the devices.

#### Measures for the intensive-longitudinal phase N-of-1: ecological momentary assessment

The subgroup of *N*-of-1 participants receive the same self-report and objective EMA measures as described for the IL phase of the main study. An additional feature is the option to capture any written or photo notes at any point in time in the app. If participants like to leave a note, they will be able to select a questionnaire in the app and answer “What was relevant for your physical activity during leisure (just now)?” by text or “Maybe a picture says more than 1000 words: show us, alternatively, what was relevant (just now) for your physical activity in your leisure-time” by taking a photo.

*N*-of-1 participants will also participate in a data-prompted, semi-structured qualitative interview [26, 27] at the end of the study. The interview questions relate to experiences with the research questions and measurement responsiveness and are conducted by a research assistant experienced in interviewing. Interviews will be audio-recorded using a digital recorder, transcribed using the program f4transkript (audiotranskription dr.dresing & pehl GmbH, Marburg, Germany) and anonymized. The program f4analyze (dr. dresing & pehl GmbH, Marburg, Germany) will be used for coding by two researchers.

### Power analysis

An a priori power analysis with G*Power [76] was performed for the primary between-subject research questions on the difference between two work contexts for LTPA. At a significance level of 5%, a power of 95%, a medium effect of d = 0.5, and equal sample sizes we would require a sample size of 210 participants. Accounting for a potential dropout of 20%, this increases to a sample size of 252 participants.

A priori power analyses for examining the link between LTPA and behavior-related outcomes (i.e., low back pain) at a significance level of 5% and 95% power, result in different sample sizes, depending on the effect size of the relationship that we expect between the specific behavior-related outcome and LTPA. It ranges from an expected small effect size, resulting in 115 participants, to a medium effect size resulting in 38 participants.

A path analytic approach to test our assumptions on the links between theory-based, psychological variables and LTPA (Fig. 1) necessitates a minimum of 210 participants [77].

Regarding a priori power analyses for multilevel models this study referred to heuristics presented in Arend and Schäfer [78], who had run Monte-Carlo simulations with a minimum of 80% power for different conditions on the smallest necessary sample size. At a significance level of 5%, a minimum of 80% power, a medium intraclass coefficient, and a minimum of *n*_Level−1_ = 14 days of assessment (i.e., number of repeated measurements within persons), small correlations for the primary outcome (LTPA) with a predictor (Level-1/day-level, e.g., intention) [79] would require a minimal Level-2 sample size of *N* = 150 (i.e., number of required individuals). For medium correlations of the outcome with a predictor (Level-2/person-level, e.g., work context), a significance level of 5%, a minimum of 80% power, a medium intraclass coefficient (ICC = 0.50) and a minimum of *n*_Level−1_ = 14 days of assessment, a minimal sample size of *N* = 100 would be required. Considering a potential dropout of 20% for the larger sample size, this estimation increases to *N* = 180.

For observational *N*-of-1 studies, there is currently no gold standard for power analyses, especially as any power is person-specific depending on e.g., individual effect size or number of observations. However, it is generally recommended to use a minimum of 50 observations in order to achieve statistically relevant results [25, 80] and good estimates [81]. To account for the potential missing data of 20%, we planned for a total of 68 days in the study.

### Data analyses

Statistical analyses will be performed separately during data collection for monitoring and at the end of data collection for the main study and for the *N*-of-1 sub-sample. Descriptive statistics will be performed for all variables (Tables 1 and 2) as well as screening for plausibility, evaluation of distributional properties and outliers, and evaluation of missing data.

#### Hypothesis tests for long-longitudinal phase

Hypotheses on between-group differences will be checked with t-tests. The association between LTPA and behavior-related outcomes with respect to the lower back will be tested with regression models. Path analyses will be used to test our assumptions on the links between theory-based, psychological variables and LTPA (Fig. 1).

#### Hypothesis tests for intensive-longitudinal phase

For the IL data with measurement days nested in participants, multilevel models with daily LTPA as outcome will be calculated. Hypothesis tests for the fixed effects will inform us on the daily links between LTPA and its within- and between-person predictors. To test mechanisms from the back health behavior model (Fig. 1), we will specify within-person mediation models and test the indirect effects.

#### Hypothesis tests for intensive-longitudinal phase N-of-1

Separate analyses will be performed for the *N*-of-1 sub-sample, as single case design requires different analysis procedure. Statistical analyses will follow dynamic regression modeling [82, 83]. Structured visual analysis of quantitative variables across the 68 days will be performed, with regards to level, trend, and variability [84]. Qualitative data from the data-prompted interviews in the *N*-of-1 sub-sample will be analyzed with the Framework Method [85] to facilitate a thematic analysis. It will also enable us to integrate qualitative findings with quantitative findings from the EMA part of the *N*-of-1 study in a pragmatic approach.

### Dissemination

The study registration and study protocol are the first publications of the workHealth study. Findings of this study will be published in peer-reviewed international journals and at national and international conferences, separately for the main study and the *N*-of-1 sub-sample. Additional dissemination to the public is planned at public institutional events and via various media.

## Discussion

Individuals can play an active role in the prevention of LBP by reducing health risk behaviors, such as breaking up sedentary time, and engaging in health-promoting PA. This includes both macro-level movements like LTPA, e.g [86], and micro-level movements like adjusting and changing back posture. Currently, there is no consensus as to which physical activity (e.g., domain, intensity, duration) is most beneficial for whom (e.g., healthy individuals in different work contexts) to prevent and to treat LBP. A more detailed understanding of the relationships between LBP and different facets of physical activity and its theory-based psychological predictors is needed to inform future behavioral interventions. The *workHealth* intensive longitudinal observational study addresses this need by integrating evidence, theory and assessment methods from psychology, biomechanics and behavioral medicine.

### Strengths, challenges and limitations

One major strength of the *workHealth* study is the multimethod assessment of physical activity including back posture and mobility (i.e., objective assessments and self-reports). Using objective measures overcomes many of the challenges associated with common method variance (e.g., inflated correlations between self-reported predictors and self-reported outcomes) or recall bias. On the other hand, there are several limitations associated with the use of accelerometers including lack of usable data, non-compliance with the wearing protocol and technical failures of the instrument [87]. A major disadvantage of using accelerometers is that it cannot provide information on the type, context and purpose of PA a person has engaged in (e.g., swimming, team sport, etc.). Therefore, the workHealth study combines the PA assessments of the Move4 with self-reports of PA. Similarly, our study combines self-reports of back posture and mobility with objective measurements. For each assessment domain, we will validate the objective assessments with the self-reported data.

A further strength of the *workHealth* study lies in its shift from a pathological view towards a more salutogenic, resource-oriented understanding of LBP [88]. While previous studies have typically been limited to studying modifiable risk factors, including health risk behaviors (e.g., heavy lifting, prolonged sitting) and health-limiting cognitions (e.g., fear of movement, pain catastrophizing), *workHealth* examines these in combination with resources such as health-promoting types of PA and self-efficacy. Furthermore, rather than looking only at the individual, the workHealth study has broadened the view to include the environment in which the individual is located, with a particular focus on the immediate workplace of two unique occupational groups: healthy, sedentary office workers and physiotherapists. Studying LBP in clinically “normal”, healthy individuals comes with methodological challenges, that is, reduced variance in the outcome. In line with the idea that the primary prevention of low back pain is not only aimed at the avoidance or reduction of LBP, but also at the opposite, the promotion of the health of the lower back – the workHealth study, therefore, uses a measure of self-reported health with regard to the lower back in addition to pain ratings.

The workHealth study is based on theory (Fig. 1) which provides specific testable predictions not only for psychological predictors contributing to LTPA, pain, and back health, but also their temporal alignment in the behavior change process. This provides important information for the development of behavioral interventions. Besides a traditional longitudinal design, the workHealth study employs an intensive longitudinal research design with daily sampling of momentary experiences and behavior in daily life (as opposed to snapshot assessments in the laboratory) which allows for testing of theory-based predictors of PA, LBP and back health within individuals, thereby allowing conclusions to be drawn about within-person dynamics of psychological and behavioral risks and resources for LPB. However, these benefits come with certain risks, such as a high expected drop-out rate or increased participant burden. With the provision of individualized feedback and monetary incentives we anticipate reducing these risks.

In conclusion, despite potential risks and limitations, the knowledge gained from our observational study is intended to advance the understanding of risk factors and resources protecting against the onset of LBP and inspire the future development and evaluation of mhealth applications for behavioral approaches to both LBP prevention and back health promotion.

## Electronic supplementary material

Below is the link to the electronic supplementary material.

**Supplementary Material 1**: Supplementary-Table-1-B-PMM.pdf. *Supplementary Table 1. Back Posture*,* Movement and Mobility (B-PMM) self-report questionnaire.* English and German language items for the Back Posture, Movement and Mobility self-report questionnaire are displayed, along with their domain (back posture, back movement, back mobility) and context (general, work, leisure-time)

## Data Availability

No datasets were generated or analysed during the current study.

## Abbreviations

LBP: Low back pain
BMI: Body–Mass–Index
PA: Physical activity
LTPA: Leisure–time physical activity
pamDC: Physical activity–mediated Demand–Control model
HAPA: Health Action Process Approach
PAAM: Physical Activity Adoption and Maintenance
BHBM: Back Health Behavior Model
EMA: Ecological momentary assessment
LL: Long–longitudinal
IL: Intensive–longitudinal
T0: Time 0, baseline
T1: Time 1
T2: Time 2
T3: Time 3
MVPA: Moderate–to–vigorous physical activity
IPAQ: International Physical Activity Questionnaire
MET: Metabolic Equivalent of Task
B-PMM self-report questionnaire: Back posture, movement, and mobility self–report questionnaire
VR-12: Veterans Rand 12–Item Health Survey
HrQoL: Health–related Quality of Life
PHQ-4: Patient Health Questionnaire–4
CPGQ: Chronic Pain Grade Questionnaire
RMDQ: Roland–Morris Disability Questionnaire
PANAS: Positive And Negative Affect Schedule
