# Supplementary material for: Linking physical activity to workers’ low back pain, back health, and theory-based psychological variables: study protocol of the workHealth intensive longitudinal observational study
Source: BMC Public Health. 2025 Mar 13;25:995. doi: 10.1186/s12889-025-21873-7 (PMC11907918; doi:10.1186/s12889-025-21873-7)
Supplement: Supplementary file 1 — Supplementary Material 1: Supplementary-Table-1-B-PMM.pdf. Supplementary Table 1. Back Posture, Movement and Mobility (B-PMM) self-report questionnaire. English and German language items for the Back Posture, Movement and Mobility self-report questionnaire are displayed, along with their domain (back posture, back movement, back mobility) and context (general, work, leisure-time) [file 12889_2025_21873_MOESM1_ESM.pdf]

Additional File 1.

Supplementary Table 1

Back Posture, Movement and Mobility (B-PMM) self-report questionnaire

Item	Englisch items	Original item wording in German	Domain	Context
1	My back posture is very good.	Ich habe eine sehr gute Rückenhaltung.	Back posture	General
2	I am very flexible in the back.	Ich bin sehr beweglich im Rücken.	Back mobility	General
3	The design of my workplace allows me to maintain a comfortable back posture (e.g., sufficient space, good adjustment of the furniture, suitable furniture).	Die Gestaltung meines Arbeitsplatzes erlaubt mir eine dauerhaft angenehme Rückenhaltung (z.B. ausreichend Platz, gute Einstellung des Mobiliars, geeignetes Mobiliar).	Back posture	Work
4	When I work, I pay attention to a posture that is gentle on my back.	Wenn ich arbeite, dann achte ich auf eine rückenschonende Haltung.	Back posture	Work
5	When I work, I pay attention to a health-promoting back posture.	Wenn ich arbeite, dann achte ich auf eine gesundheitsförderliche Rückenhaltung.	Back posture	Work
6	The design of my workplace allows me to move my back comfortably at all times (e.g., sufficient space, suitable furniture).	Die Gestaltung meines Arbeitsplatzes erlaubt mir eine dauerhaft angenehme Bewegung im Rücken (z.B. ausreichend Platz, geeignetes Mobiliar).	Back movement	Work
7	I regularly change my back posture during work (e.g., leaning back in the office chair, turning the shoulders, standing up).	Ich ändere regelmäßig meine Rückenposition während der Arbeit (z.B. im Bürostuhl nachhinten lehnen, Schultern kreisen, Aufstehen).	Back movement	Work
8	My work requires me to move my back (e.g., lifting things, walking around, instruct people on physical exercises).	Meine Arbeit erfordert, dass ich meinen Rücken bewege (z.B. Dinge heben, Umhergehen, Personen zu körperlichen Übungen anleiten).	Back movement	Work
9	When I sit during my leisure-time, I pay attention to a posture that is gentle on my back.	Wenn ich in der Freizeit sitze, dann achte ich auf eine rückenschonende Haltung.	Back posture	Leisure-time
10	When I sit during my leisure-time, I pay attention on a health-promoting back posture.	Wenn ich in der Freizeit sitze, dann achte ich auf gesundheitsförderliche Rückenhaltung.	Back posture	Leisure-time
11	When I am active during my leisure-time (e.g., shopping, recreational sports), I pay attention to my back posture.	Wenn ich in der Freizeit aktiv bin (z.B. Einkaufen, Freizeitsport), dann achte ich auf meine Rückenhaltung.	Back posture	Leisure-time
12	When I am active during my leisure-time, my back is also in a lot of movement (e.g., twisting, bending, lifting).	Wenn ich in der Freizeit aktiv bin, ist auch mein Rücken viel in Bewegung (z.B. Körperdrehungen, Beugen, Heben).	Back movement	Leisure-time
13	When I sit during my leisure-time, I regularly change my back posture (e.g., on the sofa).	Wenn ich in der Freizeit sitze, dann ändere ich regelmäßig meine Rückenhaltung (z.B. auf dem Sofa).	Back movement	Leisure-time
